# Supplementary figures and images for: Natural variation in the sequestosome-related gene, sqst-5, underlies zinc homeostasis in Caenorhabditis elegans
Source: PLoS Genet. 2020 Nov 11;16(11):e1008986. doi: 10.1371/journal.pgen.1008986 (PMC7682890; doi:10.1371/journal.pgen.1008986)

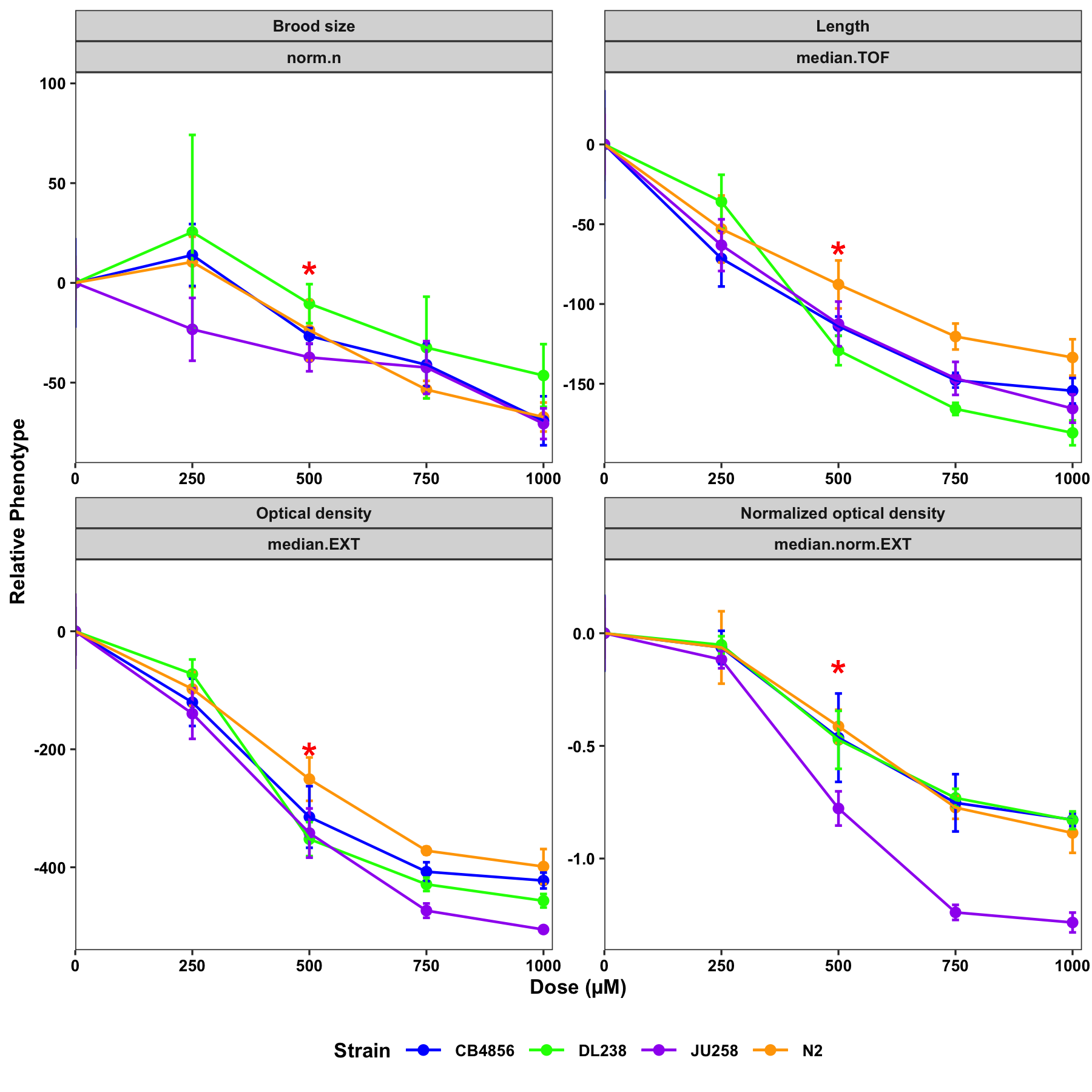

Supplement: S1 Fig — Results from the zinc dose response HTA for brood size (norm.n), animal length (median.TOF), animal optical density (median.EXT), and normalized optical density (median.norm.EXT). For each trait, drug concentration (μM) (x-axis) is plotted against phenotype subtracted from control (y-axis), colored by strain (CB4856: blue, DL238: green, JU258: purple, N2: orange). A red asterisk indicates the dose selected for linkage mapping analysis. (TIFF) [file pgen.1008986.s001.tiff]

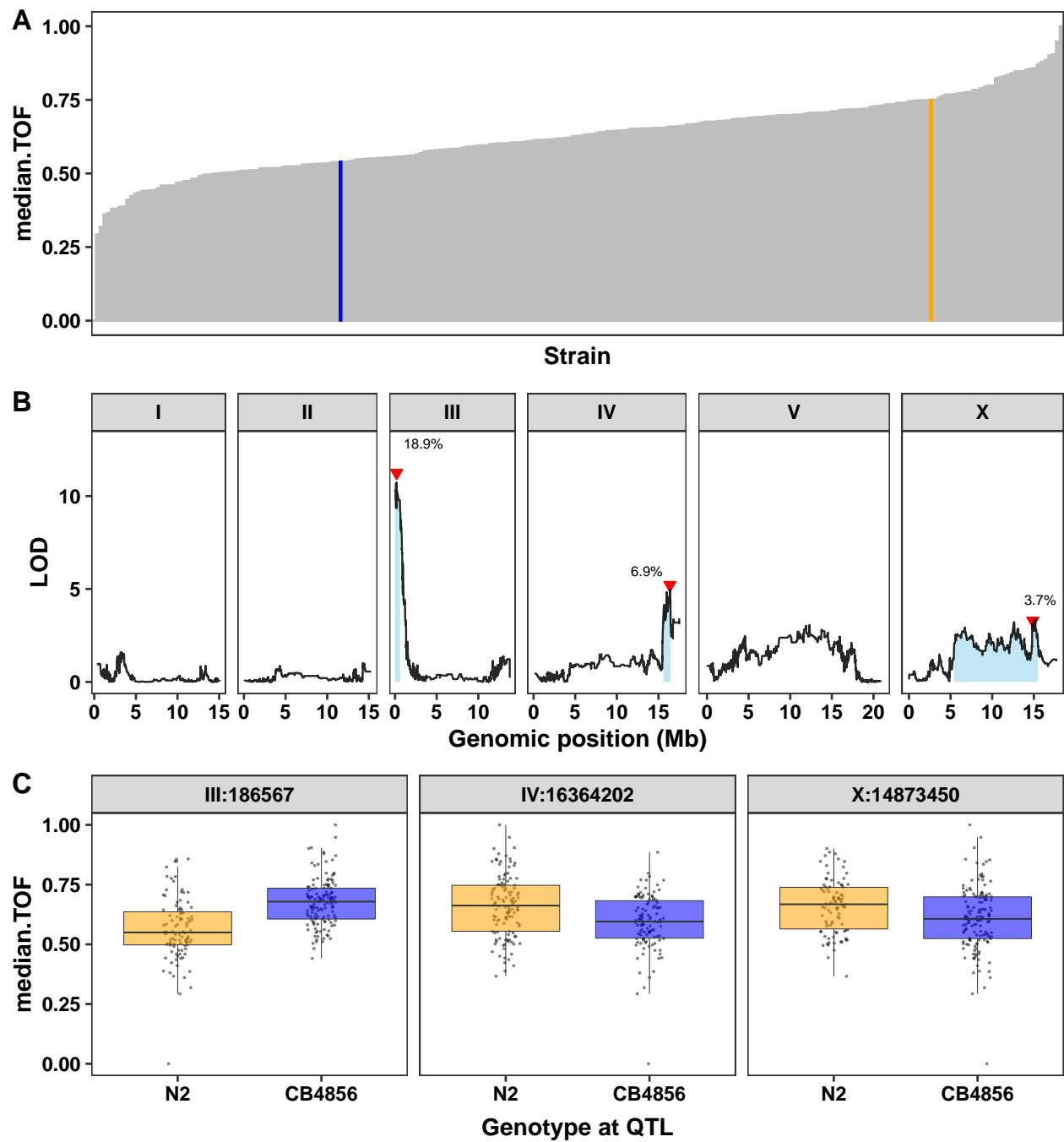

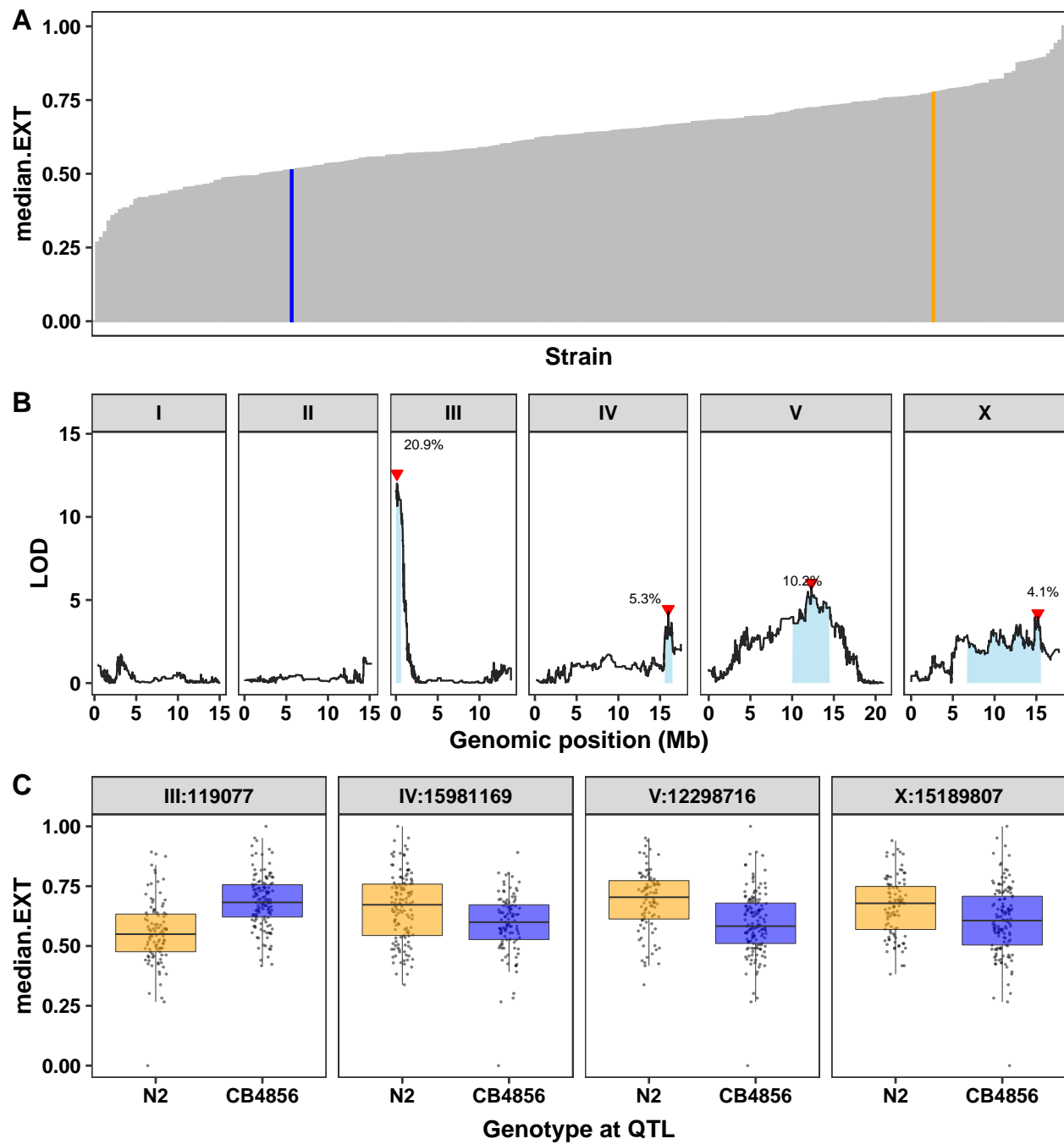

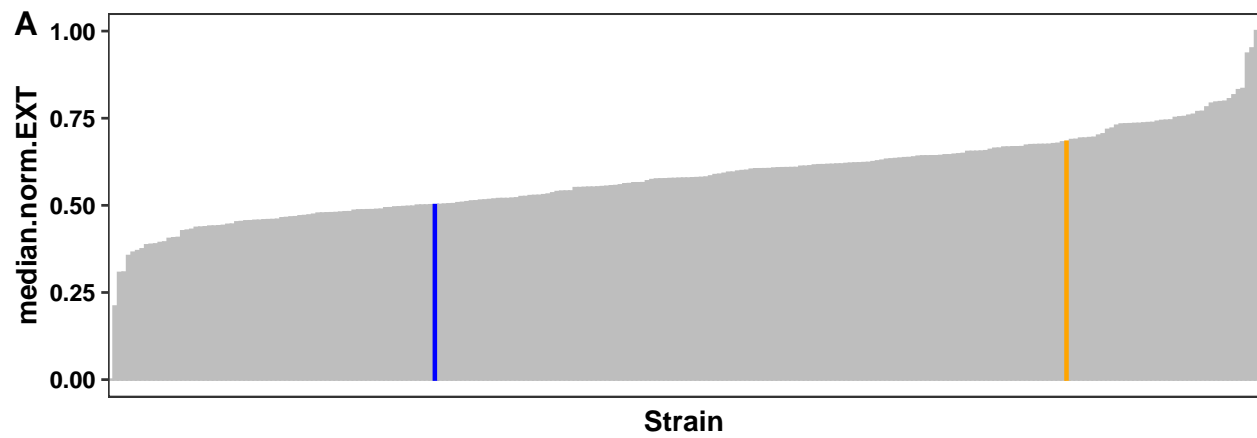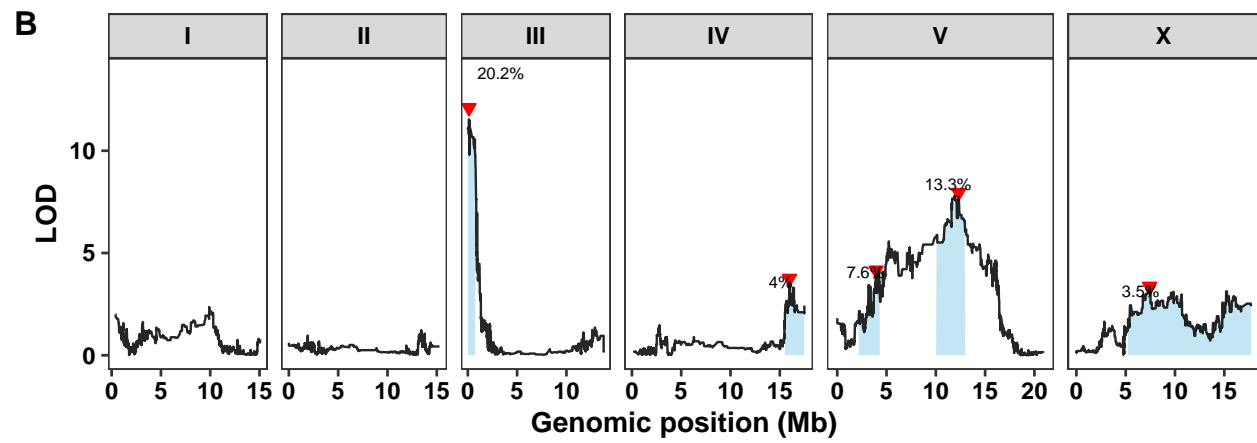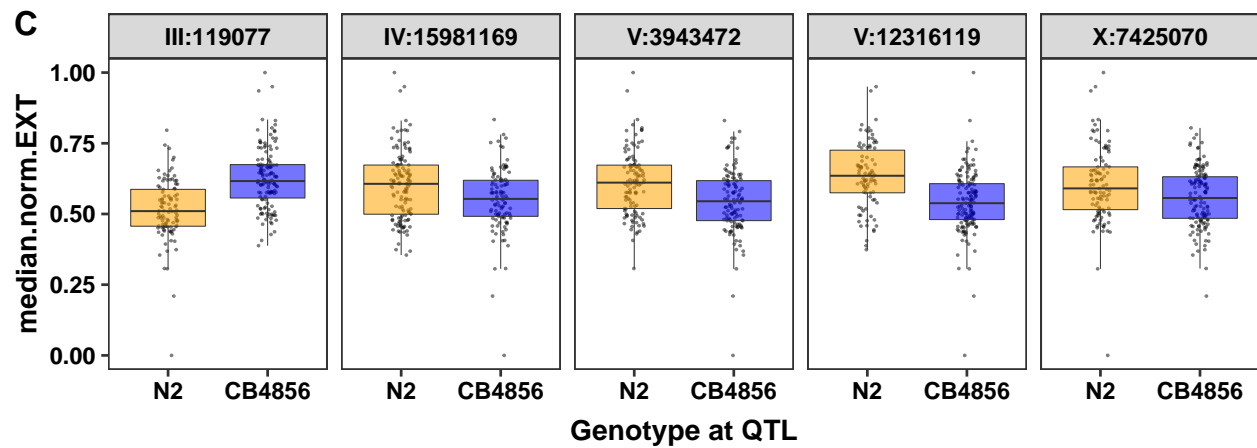

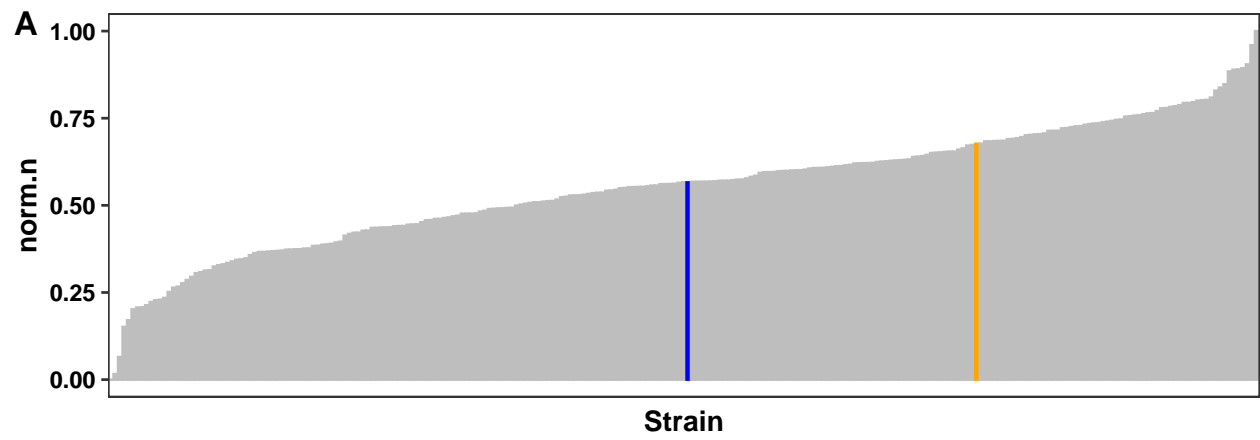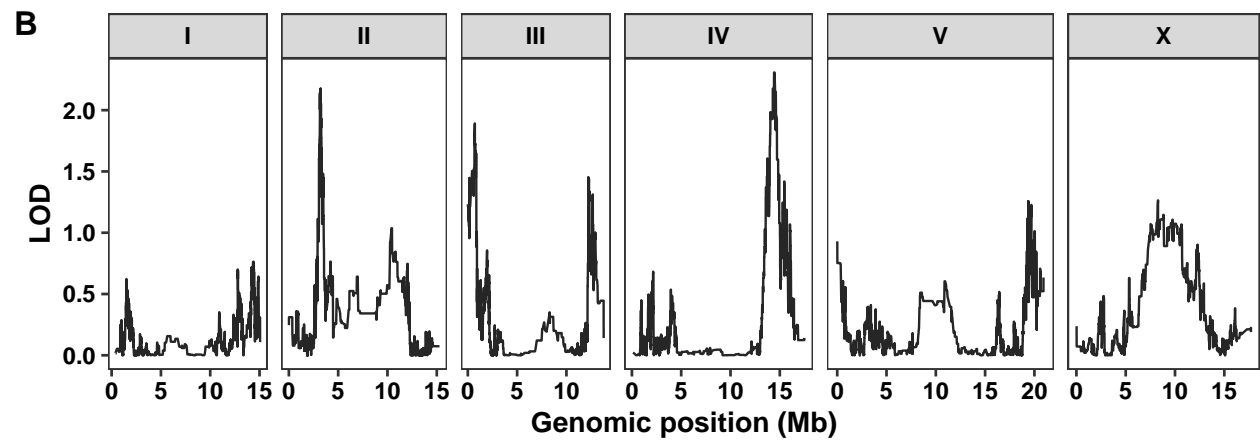

Supplement: S2 Fig — A) Normalized residual phenotype (y-axis) of 253 RIAILs (x-axis) in response to zinc supplementation. The parental strains are colored: N2, orange; CB4856, blue. B) Linkage mapping results are shown. Genomic position (x-axis) is plotted against the logarithm of the odds (LOD) score (y-axis) for 13,003 genomic markers. Each significant QTL is indicated by a red triangle at the peak marker, and a blue rectangle shows the 95% confidence interval around the peak marker. The percentage of the total variance in the RIAIL population that can be explained by each QTL is shown above the QTL. C) For each QTL, the normalized residual phenotype (y-axis) of RIAILs split by genotype at the marker with the maximum LOD score (x-axis) are plotted as Tukey box plots. Each point corresponds to a unique recombinant strain. Strains with the N2 allele are colored orange and strains with the CB4856 allele are colored blue. (PDF) [file pgen.1008986.s002.pdf]

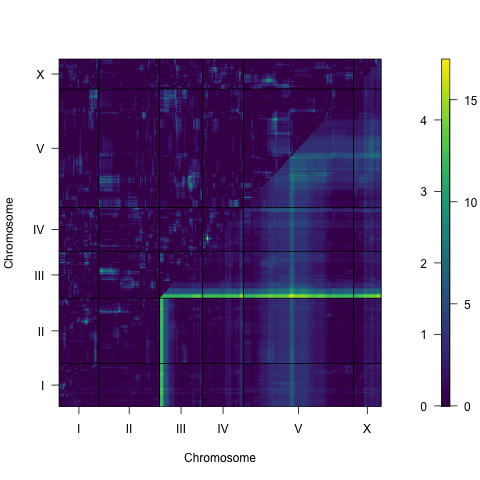

Supplement: S3 Fig — Log of the odds (LOD) scores are shown for each pairwise combination of loci, split by chromosome. The upper-left triangle contains the epistasis LOD scores and the lower-right triangle contains the LOD scores for the full model. LOD scores are colored, increasing from purple to green to yellow. The LOD scores for the epistasis model are shown on the left of the color scale and the LOD scores for the full model are shown on the right. (TIFF) [file pgen.1008986.s003.tiff]

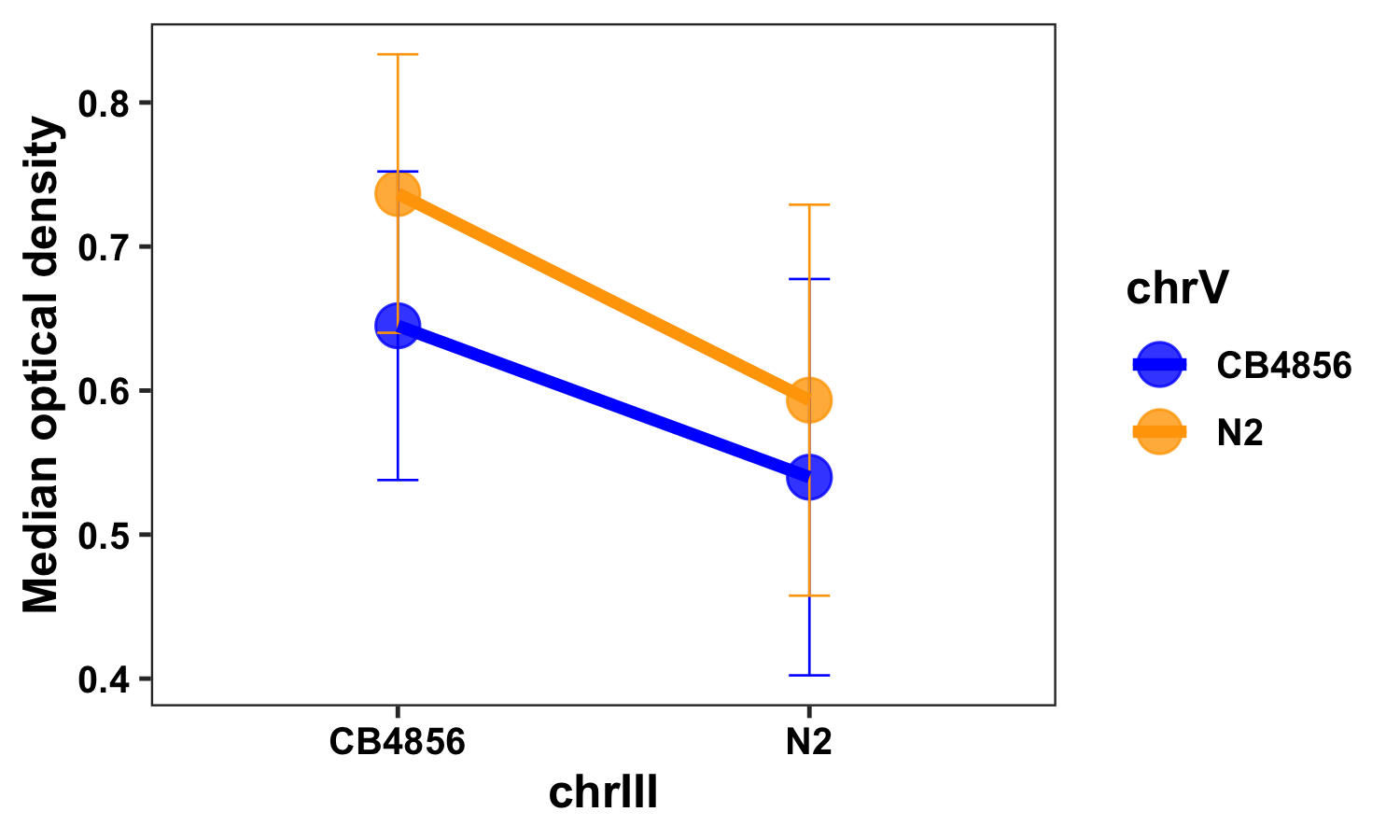

Supplement: S4 Fig — Normalized residual median optical density in zinc (median.EXT, y-axis) of RIAILs split by genotype at the chromosome III QTL (x-axis) are plotted as the mean of the population +/- the standard deviation, colored by the genotype at the chromosome V QTL. (TIFF) [file pgen.1008986.s004.tiff]

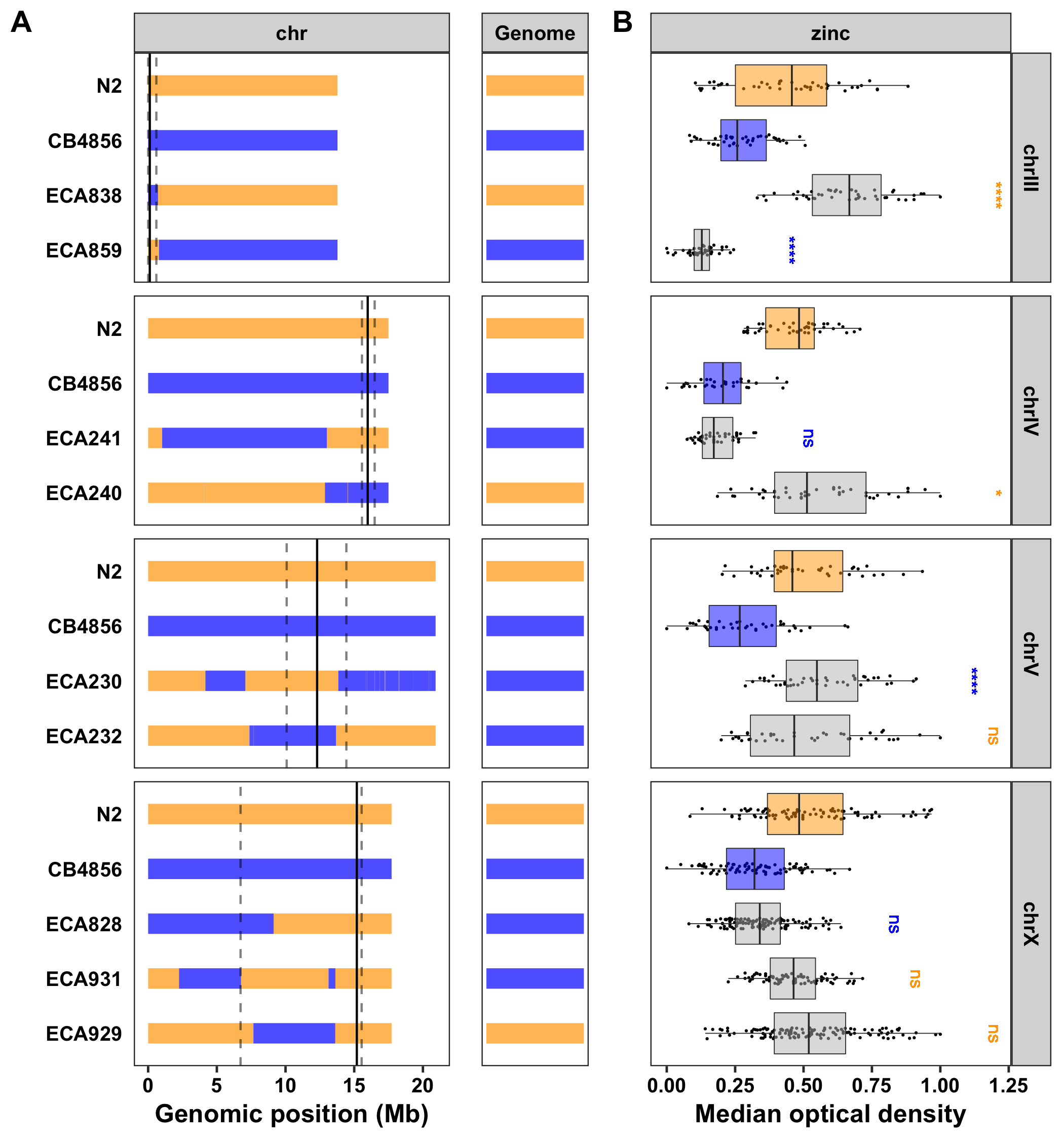

Supplement: S5 Fig — A) Strain genotypes are shown as colored rectangles (N2: orange, CB4856: blue) in detail for each chromosome (left) and in general for the rest of the chromosomes (right). The solid vertical line represents the peak marker of the QTL and the dashed vertical lines represent the confidence interval. B) Normalized residual median optical density in zinc (median.EXT, x-axis) is plotted as Tukey box plots against strain (y-axis). The parental strains N2 and CB4856 are colored orange and blue, respectively. NILs are colored grey. Statistical significance of each strain compared to its parental strain (ECA838, ECA240, ECA232, ECA931, and ECA929 to N2 and ECA859, ECA241, ECA230, and ECA828 to CB4856) is shown above each strain and colored by the parent strain it was tested against (ns = non-significant (p-value > 0.05); *, **, ***, and *** = significant (p-value < 0.05, 0.01, 0.001, or 0.0001, respectively). (TIFF) [file pgen.1008986.s005.tiff]

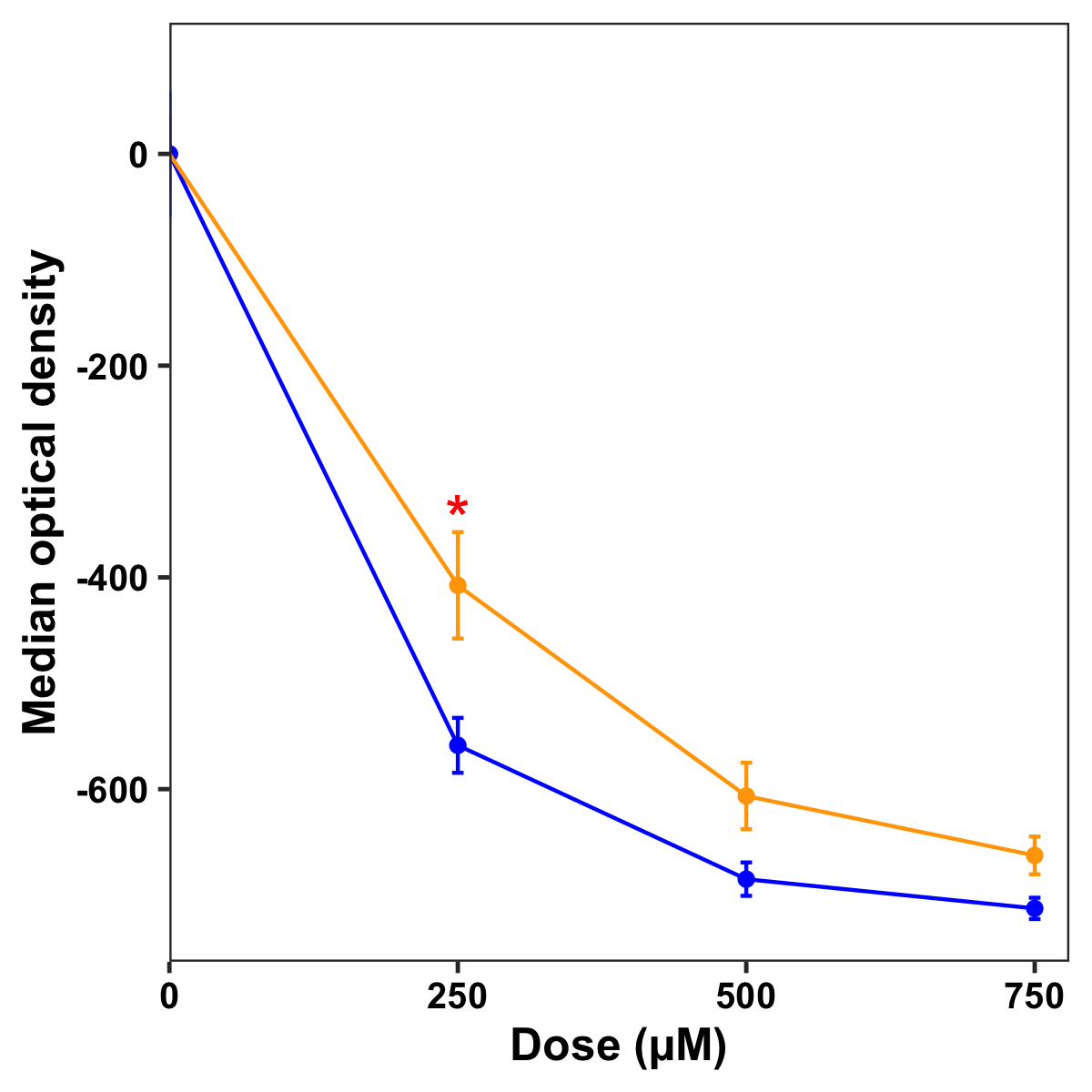

Supplement: S6 Fig — Results from the zinc dose response with the modified HTA for median optical density (median.EXT). Drug concentration (μM) (x-axis) is plotted against phenotype subtracted from control (y-axis), colored by strain (CB4856: blue, N2: orange). A red asterisk indicates the dose selected for further analysis. (TIFF) [file pgen.1008986.s006.tiff]

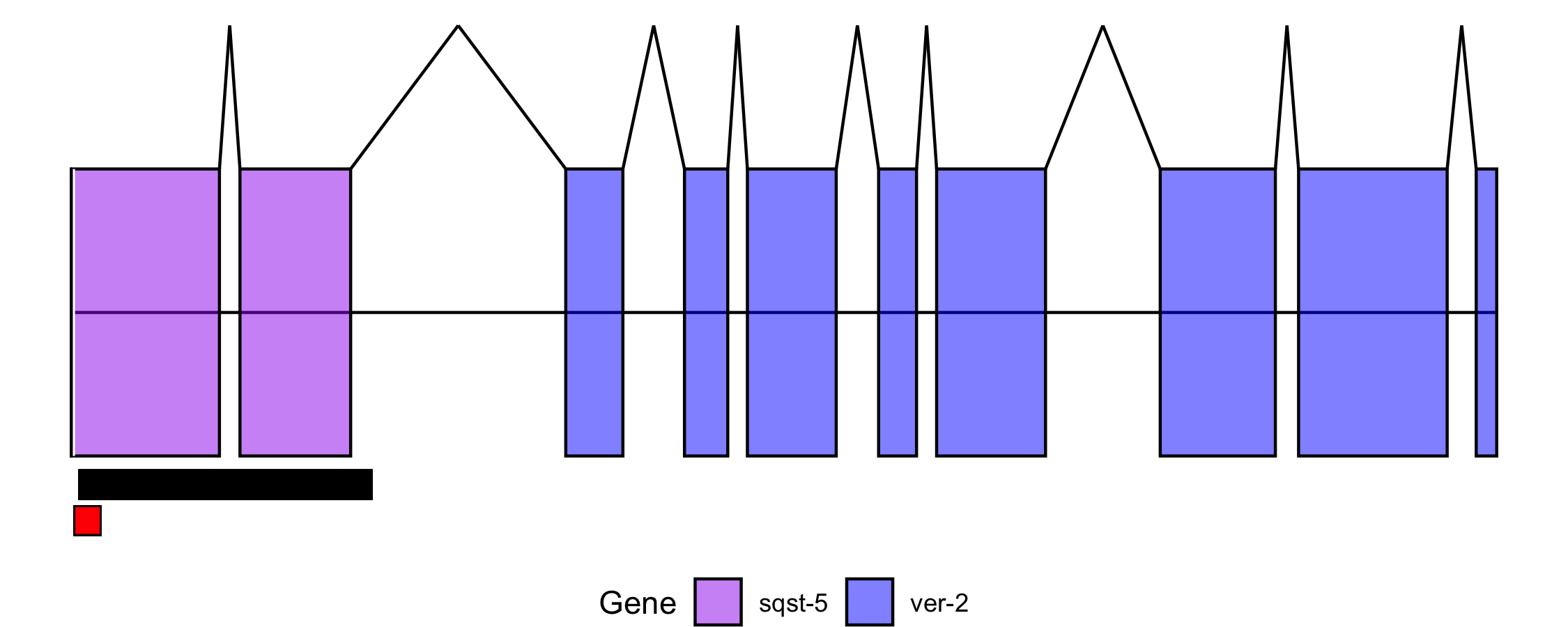

Supplement: S7 Fig — Original gene model for ver-2 is shown with colored boxes representing exons connected by lines representing introns. Exons are colored blue for the new gene model for ver-2 and purple for sqst-5. The black rectangle below represents the approximate locations of CRISPR-mediated deletions of sqst-5. The location of the microarray probe is designated as a red rectangle below the plot. (TIFF) [file pgen.1008986.s007.tiff]

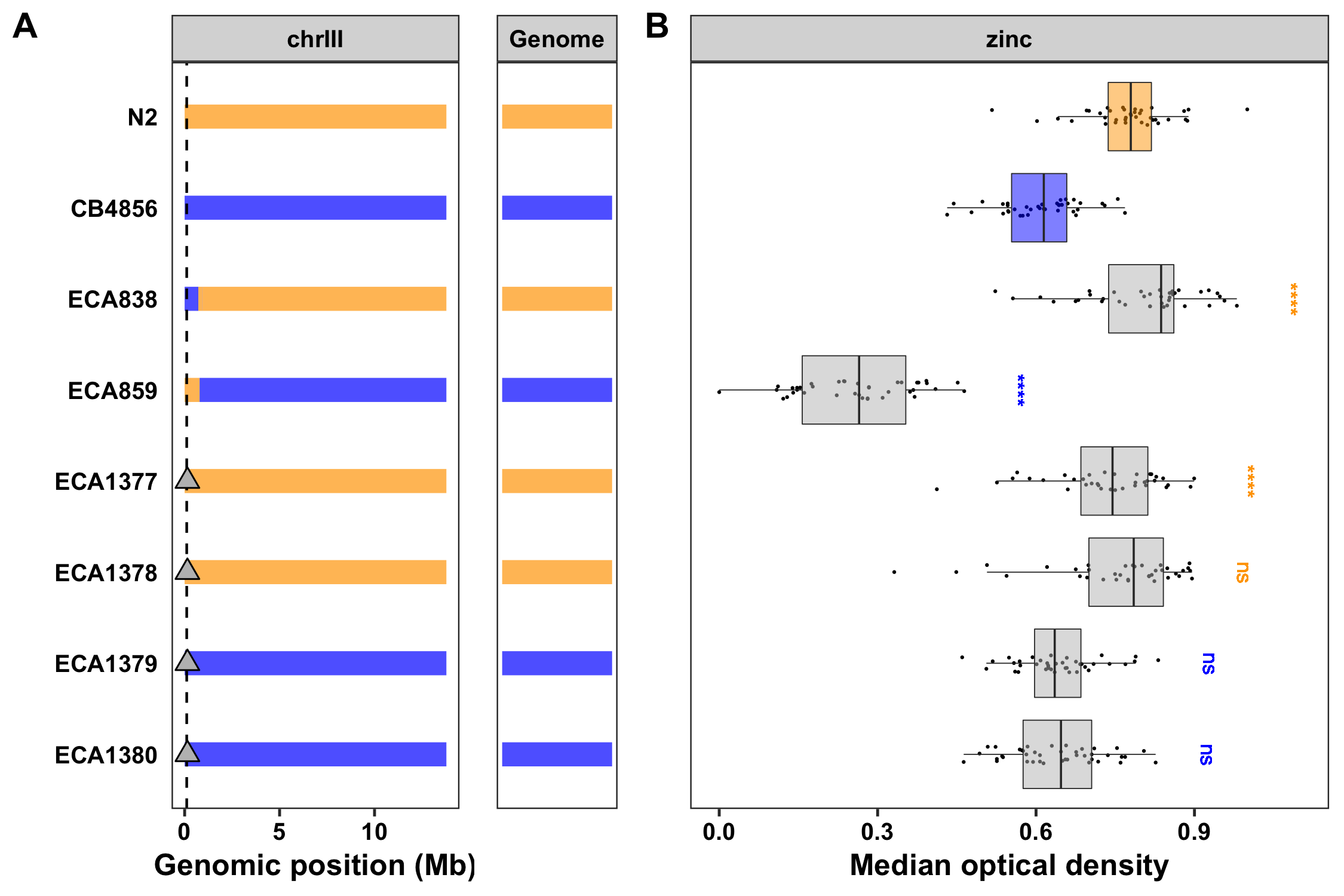

Supplement: S8 Fig — A) Strain genotypes are shown as colored rectangles (N2: orange, CB4856: blue) in detail for chromosome III (left) and in general for the rest of the chromosomes (right). The dashed vertical line represents the location of sqst-5 and grey triangles represent sqst-5 deletions. B) Normalized residual median optical density in zinc (median.EXT, x-axis) is plotted as Tukey box plots against strain (y-axis). Statistical significance of each strain compared to its parental strain (ECA838, ECA1377, and ECA1378 to N2 and ECA859, ECA1379, and ECA1380 to CB4856) is shown above each strain and colored by the parent strain it was tested against (ns = non-significant (p-value > 0.05); *, **, ***, and *** = significant (p-value < 0.05, 0.01, 0.001, or 0.0001, respectively). (TIFF) [file pgen.1008986.s008.tiff]

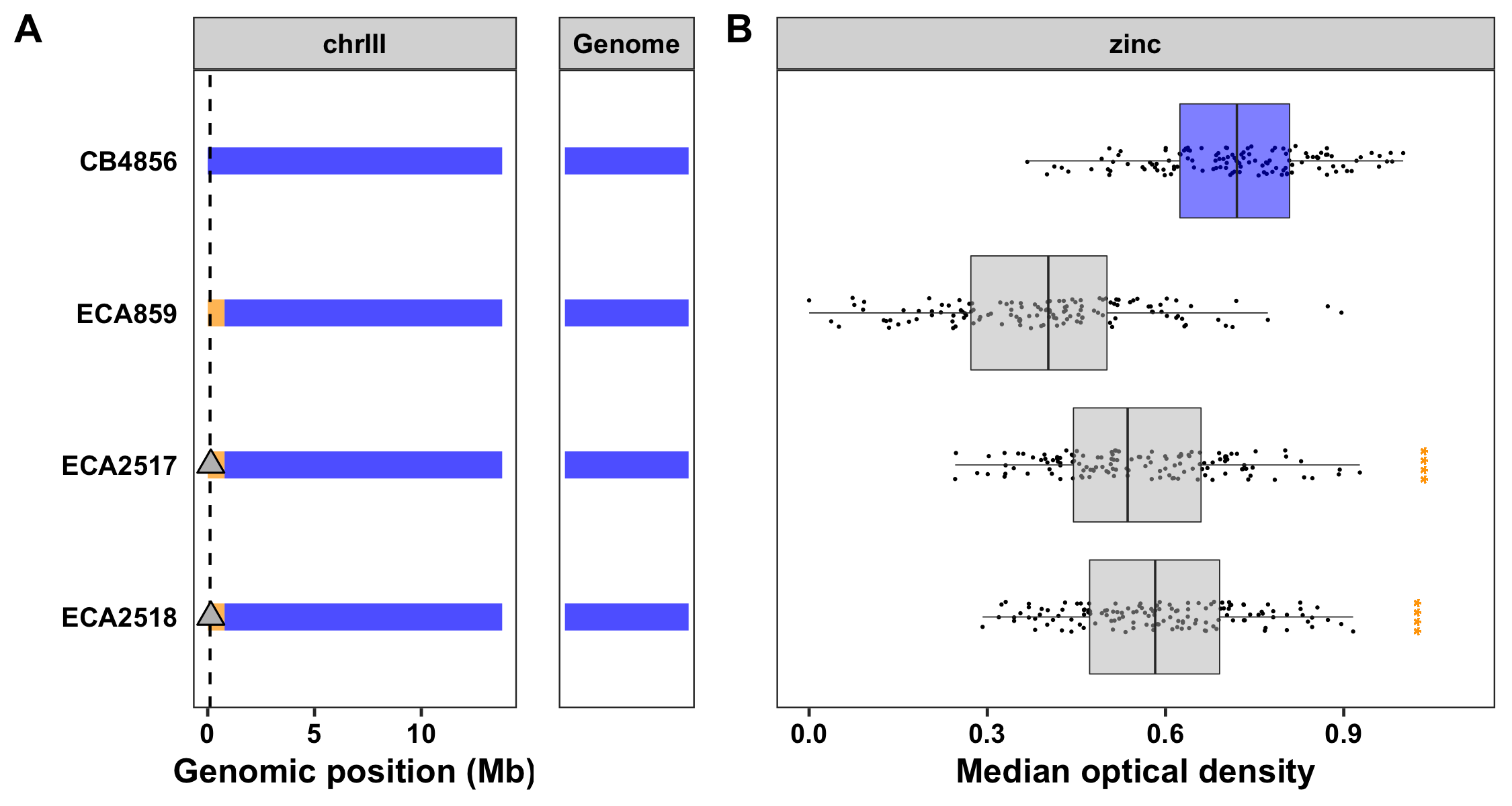

Supplement: S9 Fig — A) Strain genotypes are shown as colored rectangles (N2: orange, CB4856: blue) in detail for chromosome III (left) and in general for the rest of the chromosomes (right). The dashed vertical line represents the location of sqst-5 and grey triangles represent sqst-5 deletions. B) Normalized residual median optical density in zinc (median.EXT, x-axis) is plotted as Tukey box plots against strain (y-axis). The N2 strain, which is normally resistant to zinc, displayed an abnormal growth phenotype in this one experiment and was omitted. Because the phenotypic comparisons relevant for dominance are between NIL strains and the CB4856 strain, this omission does not affect the results. Statistical significance of each deletion strain compared to ECA859 is shown above each strain (ns = non-significant (p-value > 0.05); *, **, ***, and *** = significant (p-value < 0.05, 0.01, 0.001, or 0.0001, respectively). (TIFF) [file pgen.1008986.s009.tiff]

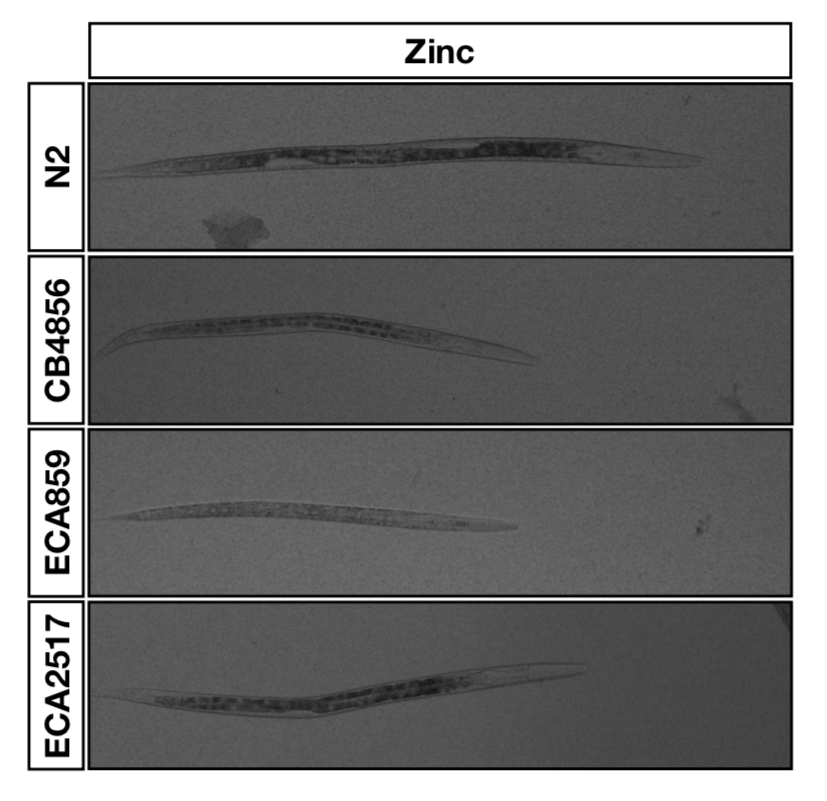

Supplement: S10 Fig — Representative images of four strains (N2, CB4856, ECA859, and ECA2517) in response to zinc are shown. (TIFF) [file pgen.1008986.s010.tiff]

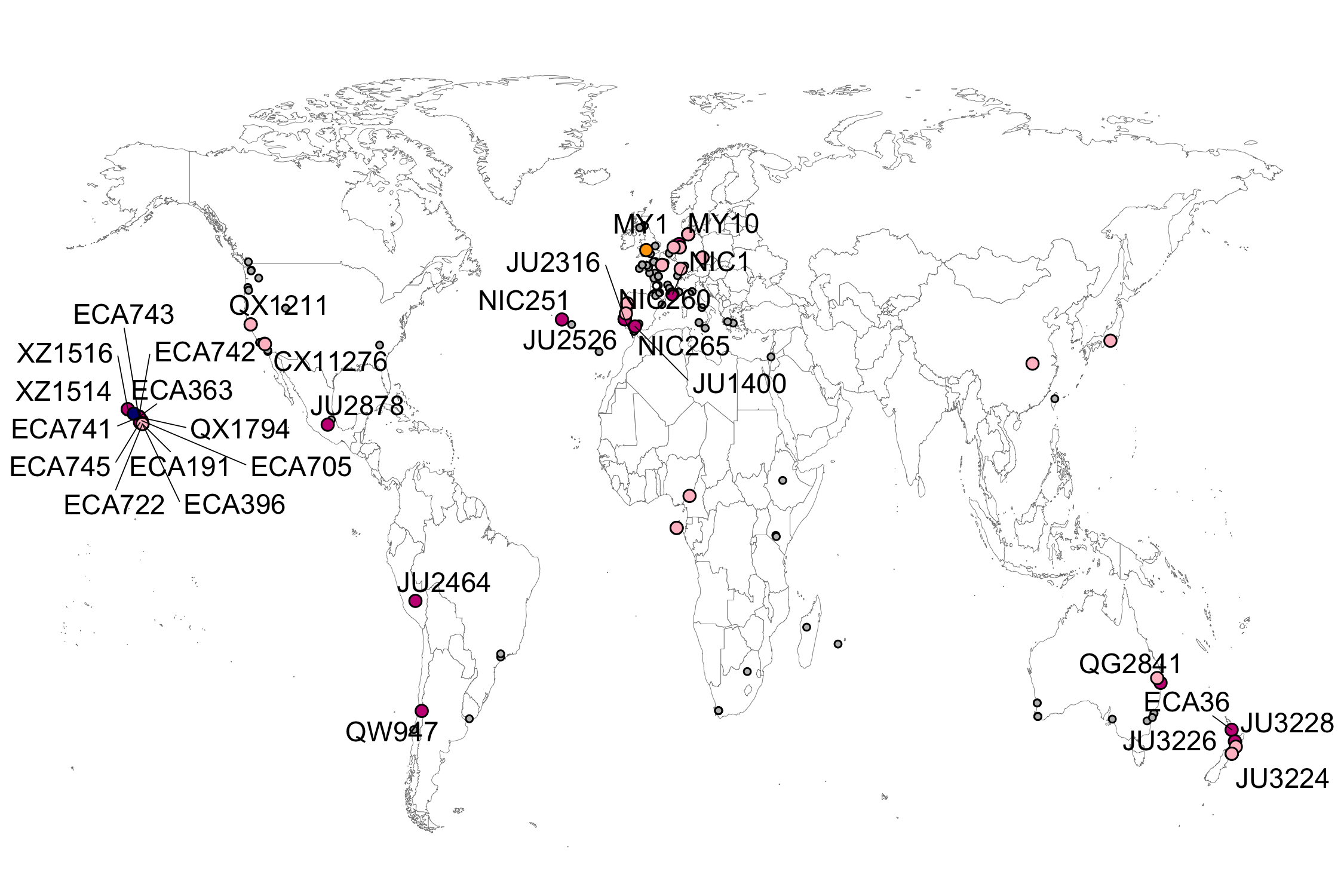

Supplement: S11 Fig — Map of wild isolates. Strains are colored by the variation haplotype at sqst-5 (Wild-type: grey, N2: orange, CB4856: navy, Deletion: magenta, Other putative structural variation: light pink). Strains with the deletion are labeled. (TIFF) [file pgen.1008986.s011.tiff]

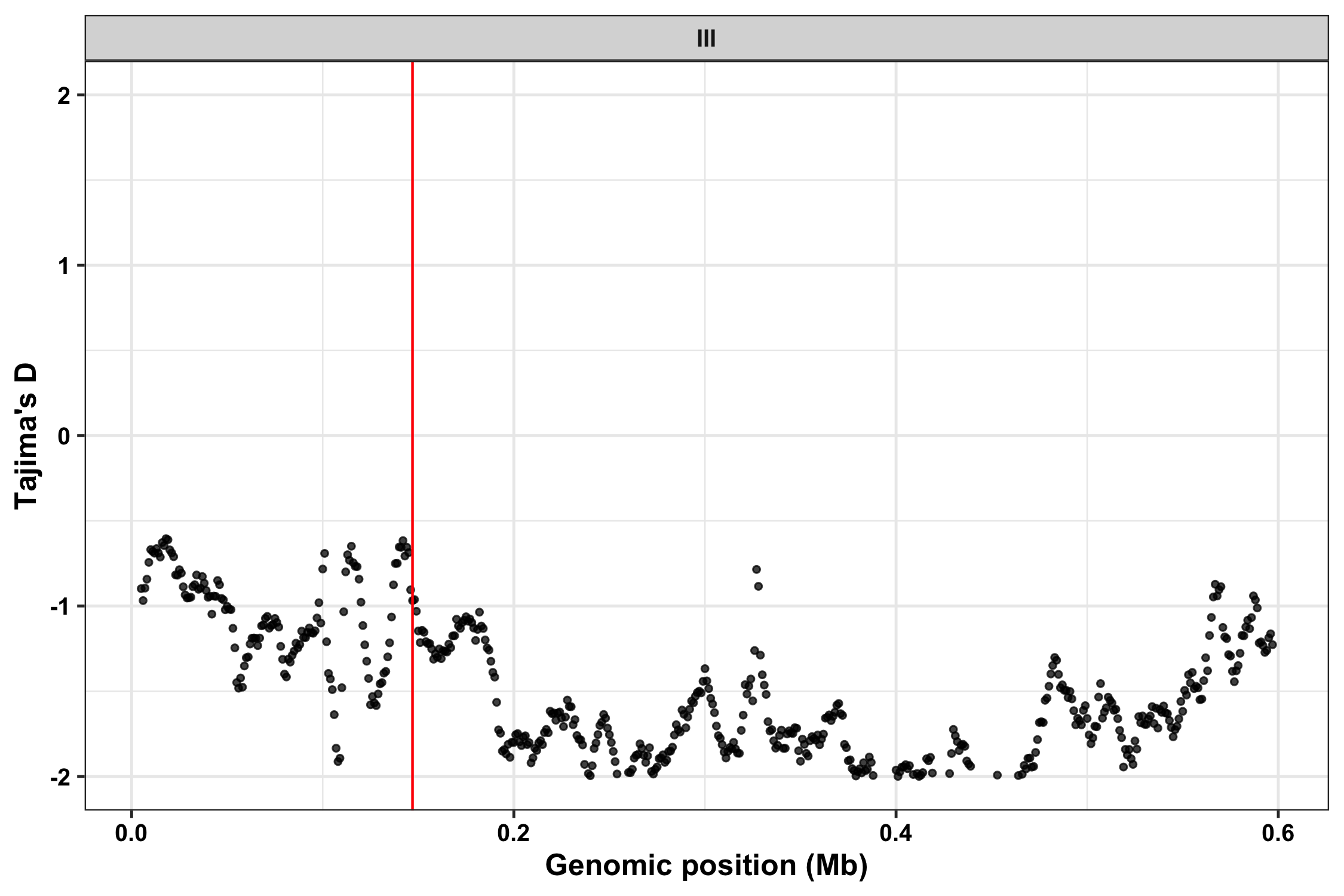

Supplement: S12 Fig — Nucleotide divergence, as measured by Tajima’s D, is shown across the zinc-response confidence interval on chromosome III (III:4,664–597,553). Window size for the calculations was 10 kb with a 1 kb sliding window size. Red vertical line represents the location of sqst-5. (TIFF) [file pgen.1008986.s012.tiff]

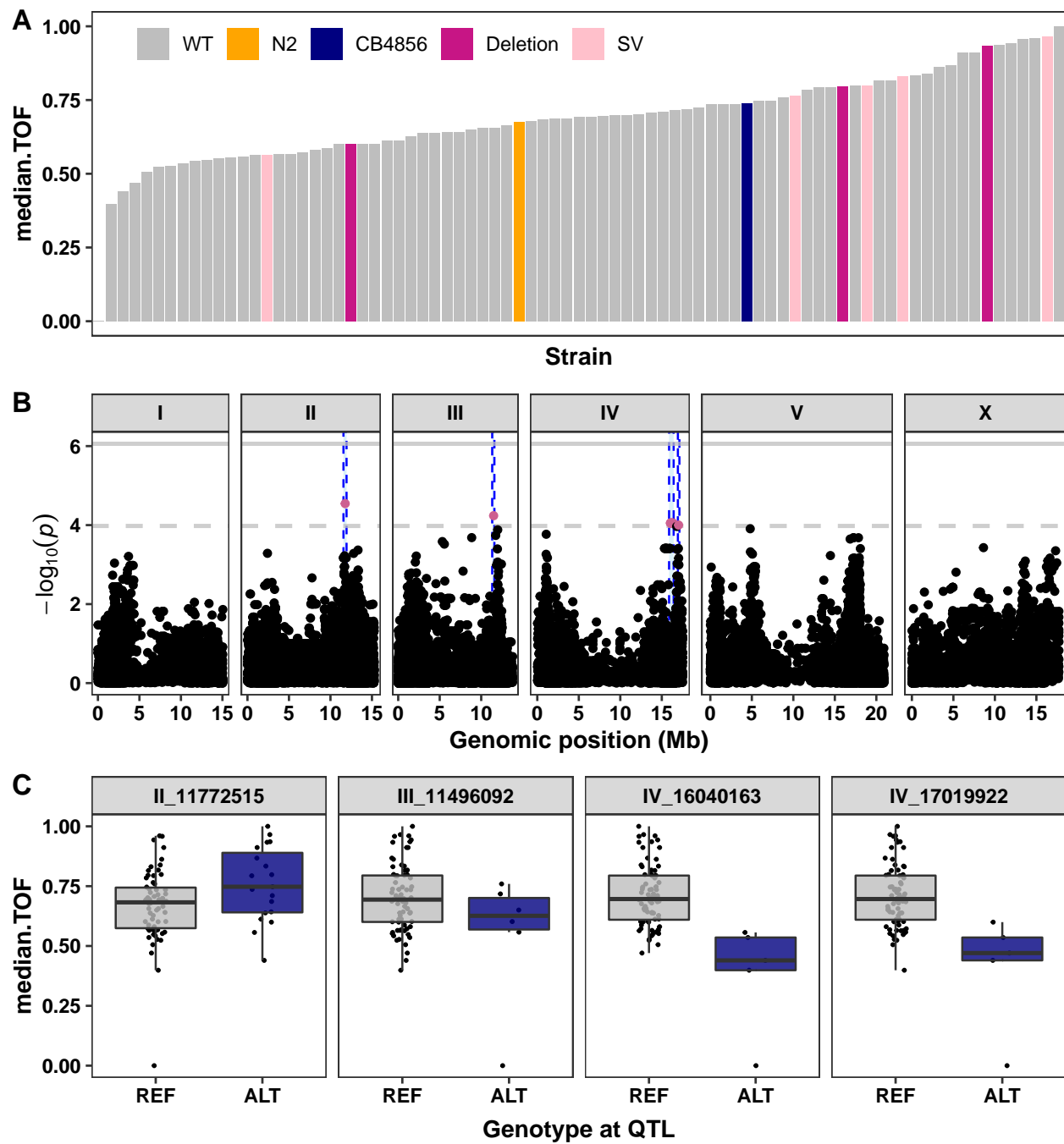

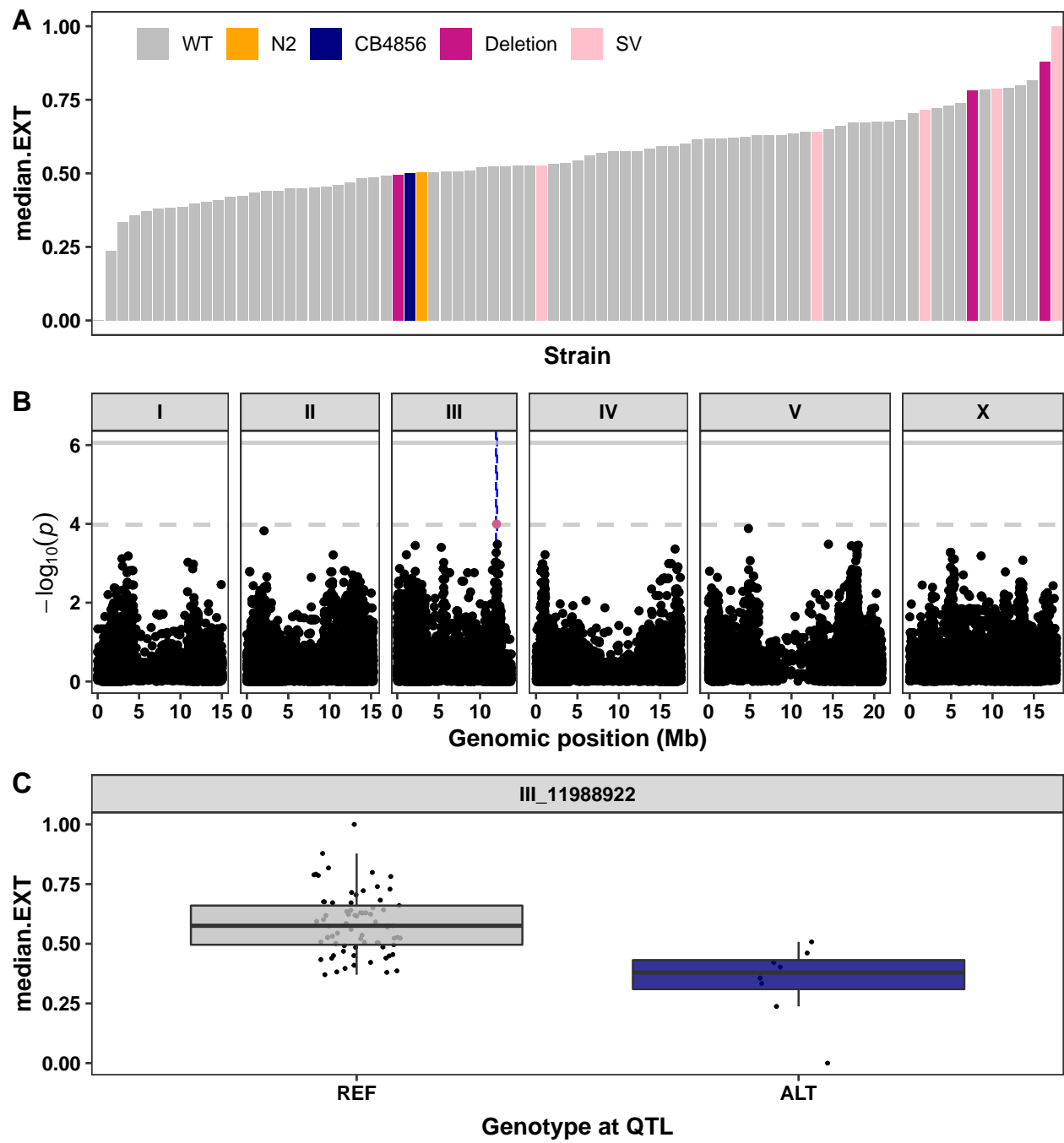

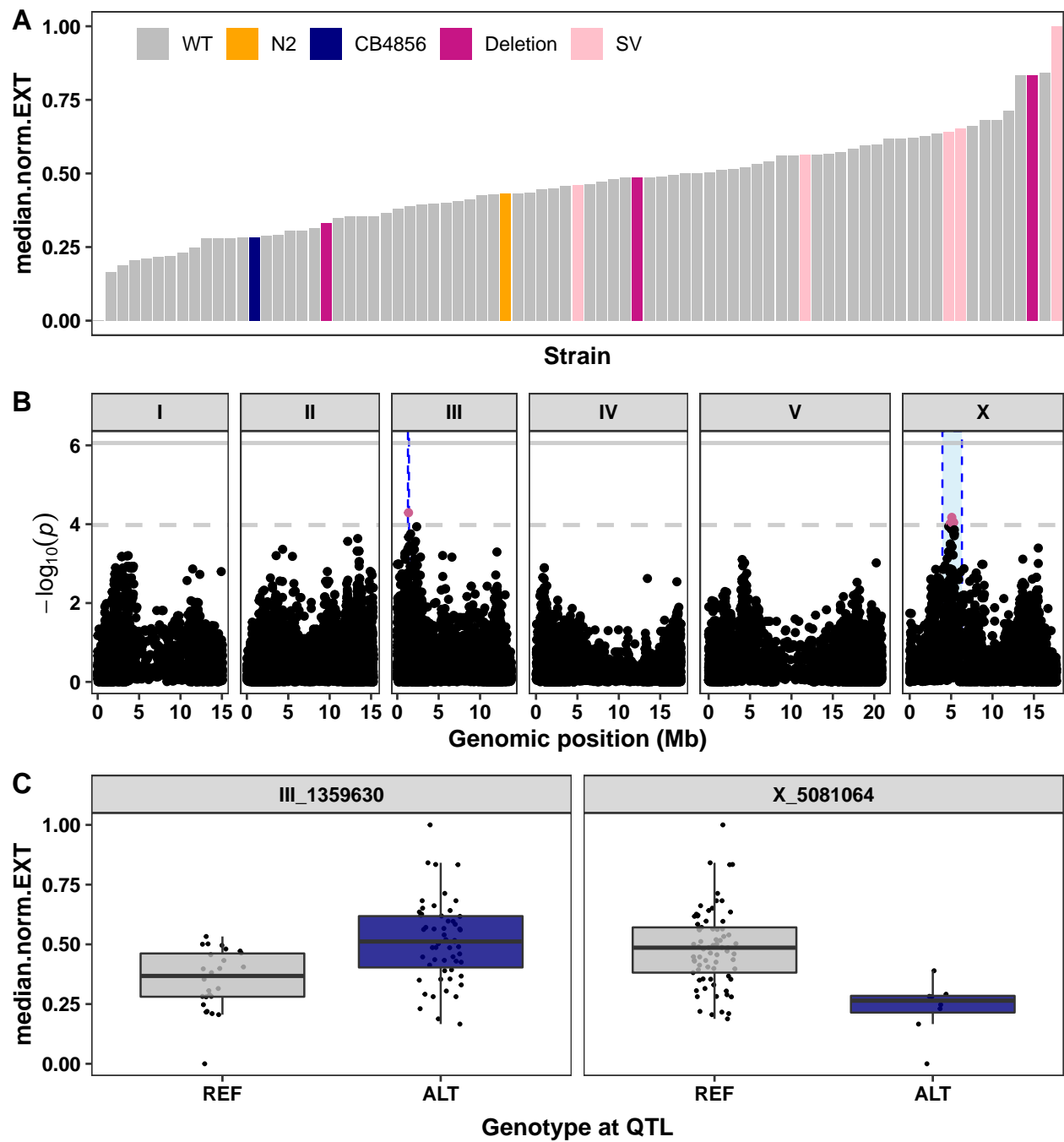

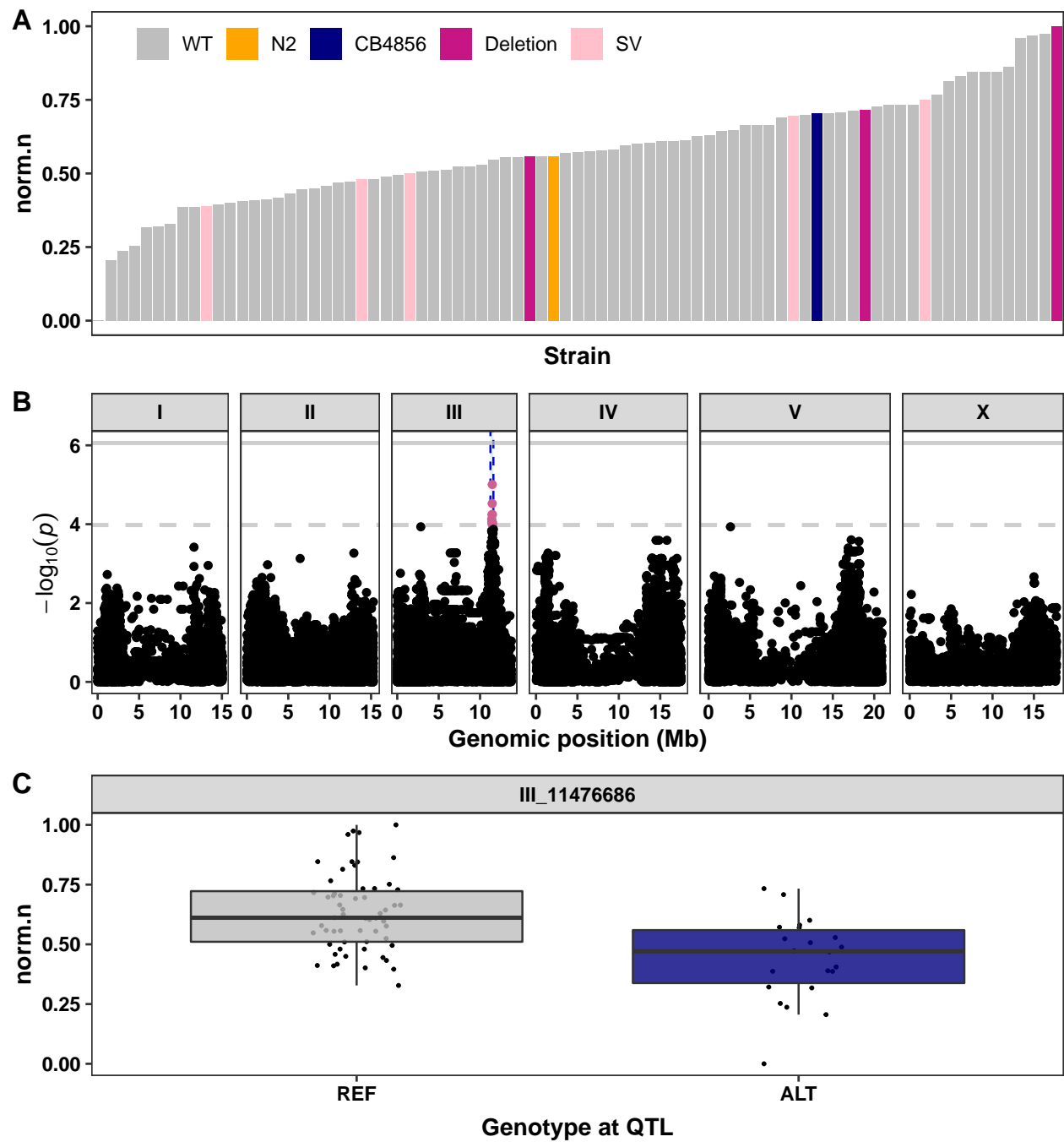

Supplement: S13 Fig — A) Normalized residual phenotype (y-axis) of 81 wild isolates (x-axis) in response to zinc supplementation. Strains are colored by the parental strains N2 (orange) and CB4856 (blue) or by the sqst-5 variation (Deletion: magenta, other variation: light pink) B) GWA results are shown. Genomic position (x-axis) is plotted against the -log10(p) value (y-axis) for each SNV. SNVs are colored pink if they pass the genome-wide eigen-decomposition significance threshold designated by the dotted grey line. The solid grey line represents the more stringent Bonferroni significance threshold. The genomic regions of interest that pass the significance threshold are highlighted by blue rectangles. C) For each QTL, the normalized residual phenotype (y-axis) of strains split by genotype at the peak marker (x-axis) are plotted as Tukey box plots. Each point corresponds to a wild isolate strain. Strains with the N2 reference allele are colored grey, and strains with an alternative allele are colored navy. (PDF) [file pgen.1008986.s013.pdf]

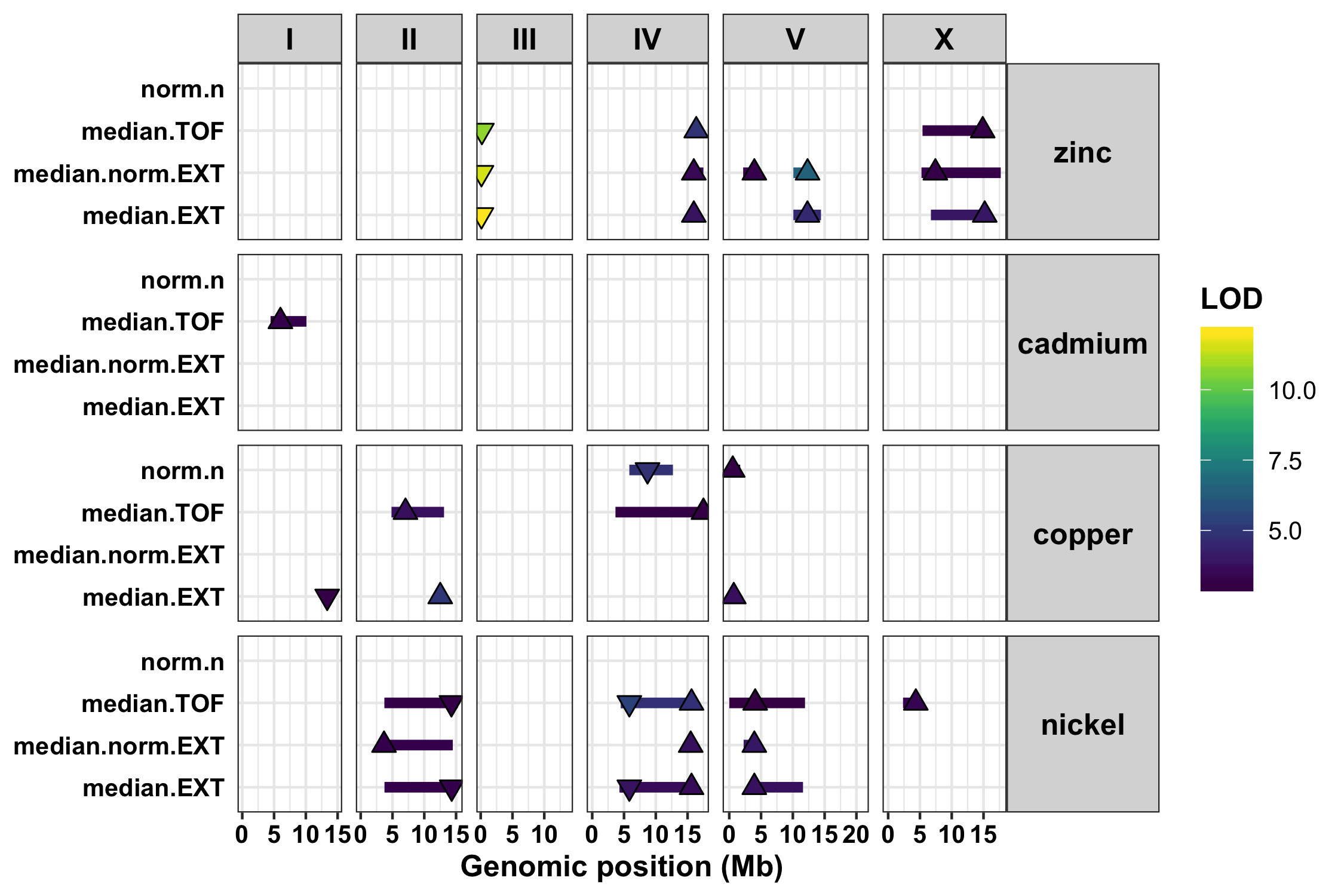

Supplement: S14 Fig — Genomic positions (x-axis) of all QTL identified from linkage mapping are shown for each drug-trait (y-axis). Each QTL is plotted as a triangle at the genomic location of the peak marker and a line that represents the 95% confidence interval. QTL with right side up triangles have a negative effect size (N2 allele is resistant), and QTL with upside down triangles have a positive effect size (CB4856 allele is resistant). QTL are colored by the logarithm of the odds (LOD) score, increasing in significance from purple to green to yellow. (TIFF) [file pgen.1008986.s014.tiff]
