# Supplementary material for: Natural variation in the sequestosome-related gene, sqst-5, underlies zinc homeostasis in Caenorhabditis elegans
Source: PLoS Genet. 2020 Nov 11;16(11):e1008986. doi: 10.1371/journal.pgen.1008986 (PMC7682890; doi:10.1371/journal.pgen.1008986)
Supplement: S7 File — VCF from the whole-genome sequencing for all the NILs in this study. (https://github.com/AndersenLab/zinc_manuscript/blob/master/data/S7_File.vcf.gz). (DOCX) [file pgen.1008986.s021.docx]

**S7 File. NIL sequence data.** <https://github.com/AndersenLab/zinc_manuscript/blob/master/data/S7_File.vcf.gz>
