# Supplementary material for: Natural variation in the sequestosome-related gene, sqst-5, underlies zinc homeostasis in Caenorhabditis elegans
Source: PLoS Genet. 2020 Nov 11;16(11):e1008986. doi: 10.1371/journal.pgen.1008986 (PMC7682890; doi:10.1371/journal.pgen.1008986)
Supplement: S1 Text — (PDF) [file pgen.1008986.s043.pdf]

Supplementary Material

List of RIAILs used:

Set 1:
QX10, QX103, QX104, QX106, QX108, QX11, QX110, QX111, QX112, QX113, QX114, QX117, QX118, QX12, QX120, QX121, QX122, QX123, QX127, QX13, QX132, QX133, QX134, QX135, QX136, QX138, QX14, QX149, QX15, QX154, QX158, QX159, QX16, QX160, QX163, QX164, QX165, QX166, QX17, QX170, QX171, QX172, QX173, QX176, QX178, QX180, QX181, QX182, QX183, QX184, QX187, QX188, QX192, QX196, QX198, QX20, QX202, QX203, QX204, QX206, QX207, QX213, QX216, QX217, QX22, QX220, QX224, QX226, QX227, QX231, QX236, QX237, QX24, QX25, QX26, QX28, QX33, QX34, QX37, QX38, QX39, QX4, QX41, QX42, QX44, QX45, QX47, QX5, QX53, QX54, QX55, QX58, QX59, QX6, QX61, QX62, QX64, QX65, QX66, QX69, QX70, QX71, QX72, QX73, QX74, QX75, QX78, QX79, QX80, QX82, QX83, QX84, QX87, QX88, QX89, QX9, QX90, QX91, QX92, QX95, QX97

Set 2:
QX240, QX241, QX242, QX243, QX244, QX245, QX250, QX252, QX253, QX254, QX258, QX261, QX263, QX264, QX265, QX266, QX269, QX270, QX272, QX273, QX274, QX276, QX277, QX278, QX280, QX282, QX283, QX284, QX285, QX286, QX287, QX288, QX290, QX291, QX293, QX294, QX296, QX297, QX298, QX301, QX302, QX304, QX305, QX307, QX309, QX310, QX311, QX312, QX313, QX315, QX316, QX318, QX319, QX320, QX321, QX323, QX324, QX325, QX326, QX327, QX328, QX329, QX330, QX331, QX332, QX333, QX334, QX336, QX337, QX339, QX340, QX341, QX345, QX346, QX347, QX348, QX349, QX352, QX353, QX354, QX355, QX356, QX357, QX358, QX359, QX360, QX361, QX362, QX363, QX365, QX366, QX367, QX368, QX369, QX370, QX372, QX373, QX374, QX375, QX376, QX377, QX378, QX379, QX380, QX381, QX382, QX383, QX384, QX385, QX386, QX387, QX389, QX390, QX391, QX392, QX393, QX394, QX395, QX396, QX397, QX398, QX399, QX400, QX401, QX402, QX403, QX404, QX405, QX406, QX407, QX409, QX411, QX412, QX413, QX414, QX416, QX417, QX418, QX419, QX421, QX423, QX424, QX426, QX428, QX429, QX430, QX431, QX435, QX436, QX437, QX438, QX441, QX442, QX443, QX444, QX445, QX446, QX447, QX448, QX449, QX450, QX451, QX452, QX453, QX454, QX455, QX456, QX457, QX458, QX460, QX461, QX463, QX465, QX467, QX468, QX469, QX470, QX472, QX473, QX474, QX475, QX476, QX477, QX478, QX479, QX480, QX481, QX482, QX483, QX484, QX485, QX486, QX487, QX488, QX489, QX490, QX492, QX493, QX494, QX495, QX496, QX497, QX498, QX501, QX503, QX506, QX508, QX511, QX512, QX513, QX514, QX515, QX517, QX521, QX523, QX524, QX525, QX526, QX527, QX528, QX529, QX530, QX531, QX533, QX534, QX539, QX542, QX545, QX549, QX550, QX551, QX553, QX554, QX555, QX556, QX559, QX560, QX561, QX564, QX565, QX570, QX572, QX573, QX579, QX580, QX583, QX584, QX587, QX588, QX594, QX596, QX597, QX598

List of wild isolates used:
AB1, CB4852, CB4854, CB4856, CB4857, CB4858, CB4932, CX11262, CX11264, CX11271, CX11276, CX11285, CX11292, CX11307, CX11314, CX11315, DL200, DL226, DL238, ED3005, ED3011, ED3012, ED3040, ED3046, ED3048, ED3049, ED3052, ED3073, ED3077, EG4347, EG4349, EG4724, EG4725, EG4946, JT11398, JU1088, JU1172, JU1200, JU1212, JU1213, JU1242, JU1246, JU1395, JU1409, JU1440, JU1491, JU1530, JU1568, JU1580, JU1586, JU1652, JU1896, JU258, JU310, JU311, JU323, JU346, JU360, JU367, JU393, JU394, JU406, JU440, JU561, JU642, JU751, JU774, JU778, JU782, JU792, JU830, JU847, KR314, LKC34, LSJ1, MY1, MY10, MY16, MY18, MY23, N2, PB303, PB306, PX179, RC301

Reagents to generate NILs:

Strain	Genotype	Constructed from	Left primer	Right primer
ECA241	eanIR161 [chrIV; N2>CB]	QX375xCB4856	oECA904 & oECA905	oECA910 & oECA911
ECA240	eanIR160 [chrIV; CB>N2]	QX349xN2	oECA904 & oECA905	oECA910 & oECA911
ECA230	eanIR150 [chrV; N2>CB]	QX131xCB4856	oECA799 & oECA800	oECA745 & oECA746
ECA232	eanIR152 [chrV; CB>N2]	QX450xN2	oECA799 & oECA800	oECA745 & oECA746
ECA828	eanIR359 [chrX; N2>CB]	N2xCB4856	oECA1313 & oECA1314	oECA1246 & oECA1247
ECA931	eanIR412 [chrX; CB>N2]	N2xCB4856	oECA1238 & oECA1239	oECA1313 & oECA1314
ECA929	eanIR411 [chrX; CB>N2]	N2xCB4856	oECA1313 & oECA1314	oECA1246 & oECA1247
ECA481	eanIR255 [chrV; N2>CB]	ECA230xCB4856	oECA799 & oECA800	oECA745 & oECA746
ECA437	eanIR211 [chrV; N2>CB]	ECA230xCB4856	oECA799 & oECA800	oECA745 & oECA746
ECA411	eanIR185 [chrV; N2>CB]	ECA230xCB4856	oECA799 & oECA800	oECA745 & oECA746
ECA838	eanIR369 [chrIII; CB>N2]	ECA637xN2	oECA653 & oECA659	oECA654 & oECA660
ECA859	eanIR390 [chrIII; N2>CB]	ECA583xCB4856	oECA653 & oECA659	oECA654 & oECA660

<u>Primers</u>

Primer	Genomic position	Sequence
oECA653	III:177,972	aaaagtcccatgcacttgcg
oECA654	III:1,802,658	gctcattcaccgggatttgc
oECA659	III:177,972	ggggaggcggattcctaatc
oECA660	III:1,802,658	aatctggctccgatcgtgtc
oECA745	V:13,110,045	tgcagaggtggagtaaccct
oECA746	V:13,110,045	ctcggtctctcccccactaa
oECA799	V:7,862,556	ttctcgctactggaacacgc
oECA800	V:7,862,556	tcaagaagcgttgggaagtct
oECA904	IV:13,207,120	aacagatactcgccgttgct
oECA905	IV:13,207,120	atttgtaccacgcgtgacct
oECA910	IV:17,356,993	gacaacgcccactacgacaa
oECA911	IV:17,356,993	acccaaccagttgagcacat
oECA1238	X:5,088,087	tcagtaccgctgtcacacac
oECA1239	X:5,088,087	cgttcctttgagcaatgggc
oECA1246	X:11,696,902	tgcggtgggacttttcttgt
oECA1247	X:11,696,902	gtcccagcatgtaaccgtct
oECA1313	X:8,038,337	gctgtgcaggactggatgta
oECA1314	X:8,038,337	tgctttctgatctgtgccgt

Reagents to generate *sqst-5* CRISPR deletion strains:

<u>crECA36 *dpy-10* guide RNA:</u>
GCUACCAUAGGCACCACGAG

<u>crECA37 *dpy-10* repair construct:</u>
CACTTGAACTTCAATACGGCAAGATGAGAATGACTGGAAACCGTACCGCATGCGGTGCCTAGGTAGCGGAGCTTCACATGGCTTCAGACCAACAGCCTAT

<u>*sqst*-5 guide RNA:</u>
crECA124: CAGTGTTCAGAAACAAAACT
crECA125: CTGATAACATAAGCGTTCTT

<u>External primers (N2):</u>
oECA1693: cggtccacggctggtATATT
oECA1652: GCAGGGGTCCTTGCTTTCTCTA

<u>External primers (CB4856):</u>
oECA1695: AGCTCATCCGCAACTTTCAT
oECA1652: GCAGGGGTCCTTGCTTTCTCTA

**Strain**	**Genotype**	**Deletion**
ECA1377	*sqst-5*(ean189)<br>[N2 background]	660bp deletion from III:146996-147655. (Exons 1-2)
ECA1378	*sqst-5*(ean190)<br>[N2 background]	656bp deletion from III:147000-147655. (Exons 1-2)
ECA1379	*sqst-5*(ean191)<br>[CB background]	656bp deletion from III:147000-147655. (Exons 1-2)
ECA1380	*sqst-5*(ean192)<br>[CB background]	656bp deletion from III:147000-147655. (Exons 1-2)
ECA2517	*sqst-5*(ean194)<br>[ECA859 background]	656bp deletion from III:147000-147655. (Exons 1-2)
ECA2518	*sqst-5*(ean195)<br>[ECA859 background]	656bp deletion from III:147000-147655. (Exons 1-2)
